# A New Investigation to Discriminate Sexes in Alive Nile Tilapia (*Oreochromis niloticus*) Using *Cyp19a1a* and *Dmrt1* Gene Expression in Tail Fin Tissues

**DOI:** 10.1007/s10126-024-10340-w

**Published:** 2024-06-28

**Authors:** Samy Y. El-Zaeem, Amr El-Hanafy, Alaa A. El-Dahhar, Ayaat M. Elmaghraby, Amany M. Hendy

**Affiliations:** 1 Animal and Fish Production Department, Faculty of Agriculture-Saba-Basha, Alexandria University, Alexandria, Egypt; 2 Nucleic Acids Research Department, Genetic Engineering and Biotechnology Research Institute (GEBRI), City of Scientific Research and Technological Applications, Alexandria, Egypt; 3 Faculty of Health Sciences Technology, Borg Al-Arab Technological University, Alexandria, Egypt

**Keywords:** *Oreochromis niloticus*, Sex determination-related genes, Tail fin, Discriminant analysis (DA), *cyp19a1a*, *dmrt1*

## Abstract

The Nile Tilapia (*Oreochromis niloticus*), a gonochoristic teleost fish with a XX/XY sex-determination system, is an ideal model for investigating gonadal sex differentiation. During gonadal differentiation, the expression of *cyp19a1a* in XX gonads and *dmrt1* in XY gonads are required for undifferentiated tissues to develop into ovary or testis. In this study, quantitative real-time RT-PCR assessed the expression of *cyp19a1a* and *dmrt1* genes in gonads and tail fin tissues. Differences in gene expression mean among sexually differentiated fish were analyzed using two-way analysis of variance (ANOVA) and validation of mixed model using discriminant analysis (DA) for morphometric traits and the gene expression in gonads and tail fin tissues used to validate and utilize them in discriminating sexes in sex-differentiated Nile Tilapia fish. The results revealed that, *cyp19a1a* gene expression in female ovaries was more significant than *dmrt1* in male testis. In the other hand, the *dmrt1* gene expression in the tail fin was higher in males than females. Both, *cyp19a1a* and *dmrt1* genes, can discriminate fish sexes by 100% by using their expression in tail fin tissues. In conclusion, the *cyp19a1a* and *dmrt1* genes could be used as a genetic marker to discriminate between the Nile Tilapia sexes, whereas used as an indicator for ovarian or testis differentiation in sexually differentiated Nile Tilapia using tail fin tissues. It is worth mentioning that this is the first investigation for using *cyp19a1a* and *dmrt1* genes from Nile Tilapia tail fin tissues in sex determination.

## Introduction

A freshwater fish Nile Tilapia (*O. niloticus*) is the third most important farmed fish after carp and salmon, and the males grow substantially faster than females. Because of its economic value as one of the world’s most important farmed fish species, its production depends on all-male monosex culture because males grow faster, and to control reproduction until harvest, there are extensive researches into the development of sex control strategies (Palaiokostas et al. 2015). Nile Tilapia is a genetic sex determination (GSD) and temperature-dependent sex determination (TSD) fish with a XX/XY sex-determination mechanism that could be affected by the high temperature (masculinizing above 32 °C) (Wang et al. 2017).

The genes that determine sex in many fish species are quite diverse. Understanding the mechanisms of sex determination in most fish species used in aquaculture, however, is difficult due to the complicated and variable nature of this mechanism (Lin et al. 2017). The Amhy gene has been discovered as a possible sex-determining gene in Nile Tilapia (*O. niloticus*), and the *amhy/amhrII* signal has been proposed to have an important role in male sex determination (Li et al. 2015). Gain- and loss-of-function investigations indicate that gsdf is essential for male differentiation in Tilapia (Kaneko et al. 2015; Jiang et al. 2016). In the presence of *sf1* (splicing factor 1), the male differentiation gene *dmrt1* activates *gsdf* expression (Jiang et al. 2016). *Dmrt1* gene directly controls *sox9b* (SRY-box transcription factor 9b) by binding to a cis-regulatory region in the *sox9b* promoter (Wei et al. 2019). Moreover, both *foxl2* and *cyp19a1a* mutant alleles exhibit female-to-male sex reversal (Zhang et al. 2017).

*Dmrt1* (*Doublesex and Mab-3* (DM)-related transcription factor1) and *amh* (anti-müllerian) genes are both important in vertebrate’s testis development. *Dmrt1* is a member of the DM domain gene family. Its duplication resulted in the medaka sex-determining *DMY* gene (Matsuda et al. 2002; Nanda et al. 2002). *Dmrt1* is structurally conserved across phyla and has an early effect on differentiating testis in non-mammalian vertebrates (Smith and Sinclair 2004). *Dmrt1* was one of the first genes elevated in Nile Tilapia XY males, becoming male-specific at later stages (Ijiri et al. 2008). During differentiation, transcriptome analysis in Nile Tilapia indicated that *dmrt1* is involved in testes differentiation and development (Tao et al. 2018). It was found in the testis of normal XY and XX males, demonstrating that this is a gene unrelated to Y chromosome whose expression is linked to testicular development (Guan et al. 2000).

*Cyp19a* (The Cytochrome P450 family 19 subfamily A) is also known as *cyp19a1*, *cyp19a1a*, and ovarian aromatase (Guiguen et al. 2010). *cyp19a1a* has a high level of expression in the ovary and a lower level of expression in the testis and brain (Dalla Valle et al. 2002). *cyp19a1a* sex-specific expression in XX gonads during early gonadal differentiation is required for ovary differentiation in Nile Tilapia (Ijiri et al. 2008).

Monosex culture includes physical separation of males and females, within-species hybridization to produce all-male fish, and artificial sex reversal using hormones. Treatment with 17-methyltestosterone included in the feed of sexually undifferentiated fry was the most common approach for all-male population production in Tilapia. Combining a sex reversal method with a breeding program to obtain broodstock that produces monosex fry following the reverse technique is one option to eliminate the hormones usage. It is important to develop YY supermales in Nile Tilapia by crossing neo-females (XY) with ordinary males (XY). The use of sex-linked DNA markers simplify the procedure by differentiating XX, XY, or YY individuals, reducing individual genotype identification through progeny testing. There is no information about gene expression of sex determination genes in tail fin tissues to determine sex in Nile Tilapia. The main objective of this work is to investigate the expression of *cyp19a1a* and *dmrt1* genes in tail (caudal) fins of sexually differentiated fish, to assess whether they could be used in future research to differentiate and determine the sex of fish.

## Materials and Methods

### Sample Collection

The experimental fish used in the present study were 24 adult fish (12 males and 12 females) (aged 3–4 months); individuals were collected from Barsiq fish farm, El-Behaira, Egypt. Briefly, at sampling time, fish were measured (total length (TL), standard length (SL), and body weight (BW)) and separated based on the external morphological difference between males and females. The fish gonads and tail or caudal fins were immediately removed by dissection, frozen in liquid nitrogen, then stored at – 80 °C until analyzed. All animal maintenance and handling procedures followed the recommendations of the Institutional Animal Care Use Committee, Alexandria University, Egypt (Alex-IACUC) review report AU: 14/20/11/01/2/9.

### Fulton’s Condition Factor (*K*)

Fulton’s condition factor (*K*) is a fish welfare indicator that enables the collection of meaningful data on growth, age, reproduction, nutrition, health, and welfare status. This factor is calculated through following the formula *K* = (100 × BW)/TL^3^, where BW represents body weight (g) and TL corresponds to total length (cm) (Gonzalez-Martinez et al. 2021).

### RNA Extraction

The Genozol Tri RNA Kit (Geneaid) was used to extract total RNA from the ovary, testis, and apical sections of tail fin clip tissues according to the manufacturer’s procedure. The RNA yield quality and concentrations were tested using a Nanodrop spectrophotometer (BioDrop, England). For each sample, the RNA concentration was standardized to 50 ng.

### RT-PCR Reaction for Genes of Interest

Topreal™ One-step RT q-PCR Kit (*SYBER Green with low ROX*) (enzynomics) is used to RT-PCR reactions performed according to the manufacturer procedure for expression analysis as well as *β-actine* gene was used as a reference gene; the reaction conditions were as follows: holding at 45 °C for 30 min (cDNA synthesis step), PCR reaction initiated at 95 °C for 10 min, followed by 95 °C for 5 s, then annealing step for 30 s for various primers *cyp19a1a*, *dmrt1a*, and *β- actine* genes that reaction repeated for 50 cycles (Table 1). The specificity of real-time PCR amplification was validated by analysis of melting curves, which ensured that only one PCR product was specifically amplified at the target size. The cycle threshold (Ct) was calculated for each replicate and final values were obtained from the average of two replicates per sample. The expression values of the studied genes were normalized using the expression values of a reference gene *β-actine*.

**Table 1:** Primer sequences and characteristics used for amplification of the studied genes in Nile Tilapia

**Name**	**Primer sequence**	**Annealing**	**Ref.**
***cyp19a1a*** **F**	CTACTTTCAGCCGTTCGGTTCAG	60 °C	Poonlaphdecha et al. (2013)
***cyp19a1a*** **R**	GTTCGGGTCTCGGAGGGTTTG		
***dmrt1*** **F**	CGGATTGCAGCGGACCGA		
***dmrt1*** **R**	GGACAGAGACACAGGACTAC		
***β-Actine*** **F**	GATATCATTTGCCTGAAACCGTTT	59 °C	Rengmark et al. (2007)
***β-Actine*** **R**	CGATTTCATCTTCCATGGCTTT		

*cyp19a1a* the Cytochrome P450 family 19 subfamily A, *dmrt1* the double-sex and mab-3-related transcription factor 1

Studied gene expression analysis was done using 2^−∆∆Ct^ method (Livak and Schmittgen 2001) according to the following equation:

Fold difference = 2^−ΔΔCt^

$$\Delta C_{t \;\text{sample}} - \Delta C_{t\; \text{calibrator}} = \Delta \Delta C_{t}$$

$$C_{t\; \text{GOI}} \,\text{s} - C_{t\; \text{norm}}\, \text{s} = \Delta C_{t \;\text{sample}}$$

$$C_{t\; \text{GOI}}\, \text{c} - C_{t \;\text{norm}}\, \text{c} = \Delta C_{t\;\text{calibrator}}$$

The ΔΔ*C*_*t*_ method is a popular methodology for comparing experimental sample data with a calibrator (e.g., untreated or wild-type sample) and a normalizer (e.g., housekeeping gene expression). With this method, *C*_*t*_ s for the gene of interest (GOI) in both the test sample(s) and calibrator sample are now normalized in relation to a normalizer (norm) gene *C*_*t*_ from the same two samples. The obtaining ΔΔ*C*_*t*_ value is incorporated to calculate the fold difference in expression.

The Ct method is an effective approach for comparing the results of experimental samples that include both a calibrator (such as an untreated or wild-type sample) and a normalizer (e.g., housekeeping gene expression). Using this approach, the Ct values for the gene of interest (GOI) in the test sample(s) and calibrator sample are now modified to the gene’s normalizer (norm) Ct values from the same two samples. The obtained Ct value is used to compute the fold difference in expression.

### Statistical Analysis

The following model was used to investigate differences in mean gene expression across sexually differentiated fish using two-way analysis of variance (ANOVA) (CoStat 2017):

$$Y_{ijl} = \mu + G_{i} + S_{j} + (GS)_{ij} + e_{ijl}$$

where *Y*_*ijl*_ is the observation of the *ijl*th parameter measured; *µ* is the overall mean; *G*_*i*_ is the effect of the *i*th gene; *S*_*j*_ is the effect of *j*th sex; (*GS*)_*ij*_ is the interaction genes by sex; *e*_*ijl*_ is the random error. Significant differences (*P* ≤ 0.05) among means were tested by the method of Duncan (1955). The *T*-test was used to analyze the morphometric characteristics.

Furthermore, the final data set was subjected to discriminant analysis (DA) which is a statistical procedure classify a collection of observations into two groups based on the values of independent, continuous categorical variables or predictors (the values of a categorical, dependent discriminant variable, also known as the grouping variable). In this study, sex (male or female) served as the categorical variable, while morphometric features and the levels of various genes’ expression acted as the predictors. The analysis produces a linear discriminant function that assigns values of the grouping variable based on the predictors’ values (Legendre and Legendre 2012). Based on the weighted combination of the independent variables, a discriminant score can be calculated according to the equation:

$$D_{i} = a + b_{1}x_{1} + b_{2}x_{2} +\dots + b_{n}x_{n}$$

where *D*_*i*_ is the predicted (discriminant) score, *a* is constant, *x* is the predictor, and *b* is the discriminant coefficient.

## Results

### Morphometric Characteristics

ANOVA test results reported that body weight (BW), total length (TL), and standard length (SL) differed significantly high (*P* ≤ 0.05) between females and males, with the mean values of these variables between 61.11 ± 14.78 and 184.736 ± 27.09, 14.2 ± 0.92 cm and 21.18 ± 1.28 cm, and 11.47 ± 0.86 cm and 17.1 ± 1.15 cm, respectively. There are no significant differences (*P* ≤ 0.05) between females and males in Fulton’s condition factor (*K*) (Table 2).

**Table 2:** Descriptive statistics of the body measurements of Nile Tilapia fish (mean ± SE)

*Parameter*	*Female*	*Male*	*P-value*
*BW*	61.11 ± 14.78	184.736 ± 27.09	0.002*
*TL*	14.2 ± 0.92	21.18 ± 1.28	0.001**
*SL*	11.47 ± 0.86	17.1 ± 1.15	0.003*
*K factor*	1.99 ± 0.12	1.86 ± 0.07	0.369^n.s^

*BW* body weight (g), *TL* total length (cm), *SL* standard length (cm), *K factor (%)* Fulton’s condition factor

**Significant at a statistical level (*P* ≤ 0.05)

^n.s^Lacking statistical significance

### The Expression Profile of *cyp19a1a* and *dmrt1* Genes

**Fig. 1:**
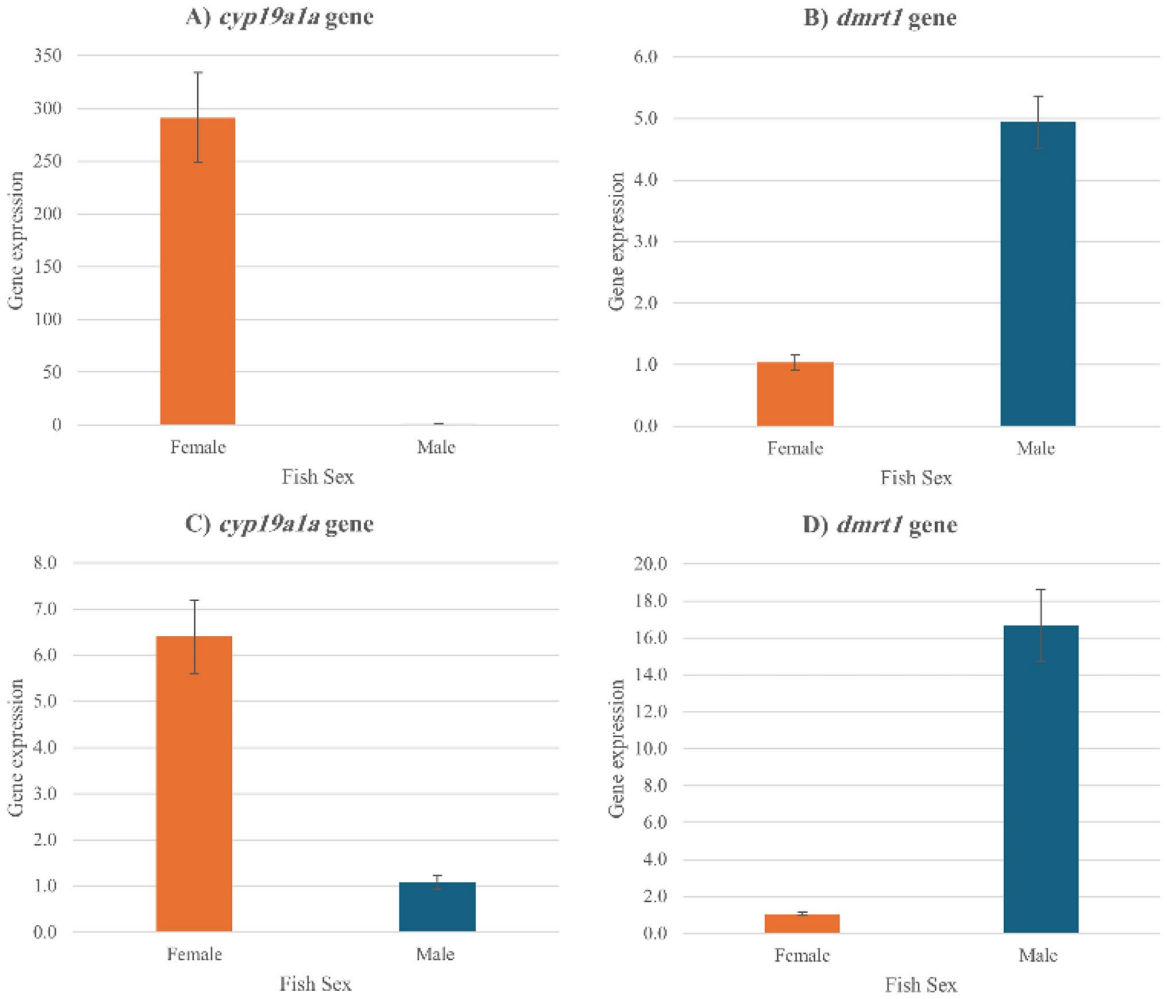
Comparative gene expression of *cyp19a1a* (**A**) and *dmrt1* (**B**) in gonads and *cyp19a1a* (**C**) and *dmrt1* (**D**) in tail fins tissues. Each value represents mean ± SE

The expression of all studied genes was detected in both gonads and tail fin tissues of females and males. Comparative gene expression analysis of sex determination genes was conducted, and the results showed that in gonads, the female ovaries had higher levels of *cyp19a1a* gene expression than in the male testis. Also, *Dmrt1* gene expression in the male’s testis was higher than in the female’s ovaries (Fig. 1A, B). In tail fin tissues, *cyp19a1a* gene expression was higher in the female’s tail fins than in the males. In contrast, *Dmrt1* gene expression which was higher in males than females (Fig. 1C, D).

### Sex-Related Differences of *cyp19a1a* and *dmrt1* Genes in Gonads

First, we analyzed the expression of *cyp19a1a* and *dmrt1* in gonads of 3–4 months sexually differentiated female and male Nile Tilapia fish. Statistical analysis showed that there was a highly significant difference (*P* ≤ 0.05) between mean gene expressions. Concerning the effect of sex on gene expression, there was a high significance between males and females. Regarding, the interaction between G × S, the results reported that *cyp19a1a* in females’ ovaries recorded a higher gene expression value than the other interactions, as shown in Table 3.

**Table 3:** The mean of gene expression affected by genes (*cyp19a1a*, *dmrt1*), sex (male and female), and their interaction in Nile Tilapia fish (*O. niloticus*) gonads and tail fins tissues

Main effect		Tissue	
Genes (G)		Gonads	Tail fins
*cyp19a1a*		146.19^a^	3.74^b^
*dmrt1a*		2.99^b^	8.86^a^
Sex (S)			
Female		146.18^a^	3.73^b^
Male		3.004^b^	8.87^a^
Interaction			
G × S			
*cyp19a1a*	Female	291.32^a^	6.47^b^
	Male	1.06^b^	1.08^c^
*dmrt1a*	Female	1.04^b^	1.06^c^
	Male	4.95^b^	16.67^a^

Means having different superscripts within column in each individual main effect and the interaction are significantly different (*P* ≤ 0.05)

*cyp19a1a* the Cytochrome P450 family 19 subfamily A, *dmrt1a* the double-sex and mab-3-related transcription factor 1

### Sex-Related Differences of *cyp19a1a* and *dmrt1* Genes in Tail (Caudal) Fins

The sex-related differences in gene expression of *cyp19a1a* and *dmrt1* in tail fin tissues were analyzed in the same fish samples, where there were significant differences (*P* ≤ 0.05) between gene expressions between sexes, and *dmrt1* was more significant than *cyp19a1a.* And, in terms of sex, males were higher than females. Also, the interaction between gene and sex reported that *dmrt1* in male tail fins had the highest gene expression value among other interactions, as shown in Table 3.

### Definition of Sex Predictor Model by Discriminant Analysis (DA)

The discriminant analysis (DA) determines which variables, including morphometric characteristics (TL, SL, *K* factor, and BW) and gene expression of two studied genes in gonads, can differentiate between sexes. We checked for which gene of two genes in tail fins best defined the groups (males and females) in Nile Tilapia.

For morphometric characteristics, the BW, TL, and SL factors significantly (*P* ≤ 0.05) contributed to differentiate sex: *F* = 16.043, 19.635, and 15.397; Wilks’ *λ* test values were 0.384, 0.337, and 0.394, and can correctly classify 83.3% of fish, respectively. However, the *K* factor had no significant and high value of Wilks’ *λ* = 0.919. as shown in Table 4.

**Table 4:** The effectiveness and sexing accuracy using individual variables in the discriminant analysis using morphometric parameters and gene expression in gonads and tail fins tissues

Variables	Wilks’ lambda (*λ*)	*F*-ratio	Males %	Females %	Total %	*P*-value
BW	0.384	16.043	83.3	83.3	83.3	0.002**
TL	0.337	19.635	83.3	83.3	83.3	0.001**
SL	0.394	15.397	83.3	83.3	83.3	0.003**
*K* factor	0.919	0.884	83.3	50	66.7	0.369^n.s^
*cyp19a1a* (G1)	0.176	46.690	100	100	100	0.000**
*dmrt1a* (G2)	0.112	79.547	100	100	100	0.000**
*cyp19a1a* (G3)	0.185	44.155	100	100	100	0.000**
*dmrt1a* (G4)	0.135	64.010	100	100	100	0.000**

*BW* body weight (g), *TL* total length (cm), *SL* standard length (cm), *K factor (%)* Fulton’s condition factor, *cyp19a1a* the Cytochrome P450 family 19 subfamily A, *dmrt1a* the double-sex and mab-3-related transcription factor 1, G1 and G2 for gonads, G3 and G4 for tail fin

**Significant at a statistical level (*P* ≤ 0.05)

^n.s^Lacking statistical significance

Also, in Table 4, all expression levels of the two genes as determined by real-time RT-PCR on samplings from gonads were used to perform the discriminant analysis (DA). The results revealed that *cyp19a1a* and *dmrt1* expression levels were significantly high (*P* ≤ 0.05) contributing to differentiate sex, where *F* = 46.690 and 79.547; Wilks’ *λ* test values were 0.176 and 0.112 and able to correctly classify 100% of fish, respectively. This concluded that *cyp19a1a* and *dmrt1* had a significant effect on discriminating sex in sexually differentiated Nile Tilapia.

The result of gene expression levels of the two genes from tail fin tissues revealed that *cyp19a1a* and *dmrt1* expression levels are significantly high (*P* ≤ 0.05) contributing to separate sex, where Wilks’ *λ* test values were 0.185 and 0.135 and were capable to correctly classify 100% of the female fish, respectively (Table 4). Wilks’ *λ* values range from 0 to 1.0, the smaller the *λ* value, the more the variable discriminates well among groups. This statistically concluded that *cyp19a1a* and *dmrt1* were best for discriminating sex in sexually differentiated Nile Tilapia using tail fin tissues.

## Discussion

Owing to sex growth differences, it is generally advantageous in aquaculture to produce fish monosex populations, for example, male Nile Tilapia grow faster than females (Chakraborty and Banerjee 2010). Nile Tilapia is a genetic sex determination (GSD) plus temperature-dependent sex determination (TSD) fish, with a XX/XY sex-determination system (Wang et al. 2017). Even so, understanding the mechanisms behind sex determination in most fish species used in aquaculture is difficult due to the complicated and varied nature of these processes; we investigated the differences in morphometric parameters and gene expression of *cyp19a1a* and *dmrt1* in gonads and tail fin tissues between Nile Tilapia females and males.

While the gene expression of sex determination genes becomes less accurate in tissues not directly implicated in reproduction, we detected *cyp19a1a* and *dmrt1* expression in female and male tail fins besides gonads; these findings along with Hofsten and Olsson (2005) who reported that *Sox9a* (a gene that plays an important role in sex determination) are present in zebrafish (*Danio rerio*) pectoral fin. Our results reported a high expression of the *cyp19a1a* gene in female gonads which is consistent with previous research on the Southern flounder (Luckenbach et al. 2005), Atlantic halibut (Matsuoka et al. 2006), and rainbow trout (Vizziano et al. 2007) which suggested that the expression of the *cyp19a1a* gene acts as an early marker of sex differentiation in these species. Furthermore, this research, combined with studies on Nile Tilapia conducted by (Ijiri et al. 2008) demonstrated that *cyp19a1a* in XX gonads during early gonadal differentiation is crucial for ovary differentiation. In males’ tail fins unlike gonads, we found a higher expression of the *dmrt1* gene than *cyp19a1a*. These findings were consistent with the research of (Guan et al. 2000) who discovered that the *dmrt1* gene is not linked to the Y chromosome, whose expression is correlated with testicular development because it was expressed in the testis of normal XY males and XX males. It is likely to Panagiotopoulou et al. (2023) who found genetic sex markers in Siberian (*Acipenser baerii*) and Atlantic (*A. oxyrinchus*) sturgeons; however, they utilized a different technique, extracting genetic material from female and male tail fins.

Discriminant analysis is used in a variety of research fields, including numerical ecology applied to fisheries management to classify the manipulation of a particular ecosystem (Tudela et al. 2005); in microbiology, to differentiate between human and animal sources of contaminates in surface waters (Kaneene et al. 2007); in forensics, to estimate the sex of unknown skeletal remains (Kemkes-Grottenthaler 2005); also in medical research, for instance to discriminate between different types of anemia (Ahluwalia et al. 1995); and for the first time, it used in study sex differentiation of seabass (Blázquez et al. 2009).

In this study, discriminant analysis was used first to define which variables among morphometric parameters and genes (*cyp19a1a* and *dmrt1*) could discriminate sex in adult Nile Tilapia fish gonads and then in tail fins. Wilks’ *λ* values range from 0 to 1.0; the smaller the *λ* value, the more the variable discriminates well among groups. For morphometric characteristics, the results reported that BW, TL, and SL contributed to differentiate sex: *F* = 16.043, 19.635, and 15.397; Wilks’ *λ* test values were 0.384, 0.337, and 0.394, respectively, and could discriminate between females and males by 83.3%. This is agreed with the fact that males grow faster than females (Chakraborty and Banerjee 2010) and with Blázquez et al. (2009) who indicate that SL can classify 75.7% of European seabass fish. For gene expression, when using the expression levels of the two genes from gonads and tail fin tissues, the result revealed that *cyp19a1a* and *dmrt1* expression levels are significantly high (*P* ≤ 0.05), and these two genes contribute to a 100% accurate discrimination and classification of sexes. The research on seabass fish conducted by (Blázquez et al. 2009) revealed that the cyp19a1a gene was able to correctly classify 100% of the fish.

In conclusion, *cyp19a1a* and *dmrt1* genes could be used as a genetic marker to discriminate between the fish sexes and as an indicator for ovarian or testis differentiation in sexually differentiated Nile Tilapia. Additionally, the tissues from tail fins had significant levels of *cyp19a1a* and *dmrt1* gene expression. Therefore, they could be utilized in future studies to distinguish between various fish sexes and to identify their sex using molecular markers with the advantage that it is an un-scarified method. The use of sex-linked DNA markers could shorten the process by distinguishing XX, XY, or YY individuals in breeding programs of fish stocks, thus avoiding the identification of individual genotypes by progeny testing. Moreover, this work is the first investigation for using *cyp19a1a* and *dmrt1* genes from Nile Tilapia tail fin tissues in sex determination.

## Data Availability

No datasets were generated or analyzed during the current study.
